# Molecular Mechanistic Pathways Targeted by Natural Antioxidants in the Prevention and Treatment of Chronic Kidney Disease

**DOI:** 10.3390/antiox11010015

**Published:** 2021-12-22

**Authors:** Mohamed Mohany, Mohammed M. Ahmed, Salim S. Al-Rejaie

**Affiliations:** Department of Pharmacology and Toxicology, College of Pharmacy, King Saud University, Riyadh 1145, Saudi Arabia; mmahmed114@yahoo.com (M.M.A.); rejaie@ksu.edu.sa (S.S.A.-R.)

**Keywords:** chronic kidney disease (CKD), diabetic nephropathy, renal fibrosis, natural compounds, mechanistic pathways

## Abstract

Chronic kidney disease (CKD) is the progressive loss of renal function and the leading cause of end-stage renal disease (ESRD). Despite optimal therapy, many patients progress to ESRD and require dialysis or transplantation. The pathogenesis of CKD involves inflammation, kidney fibrosis, and blunted renal cellular antioxidant capacity. In this review, we have focused on in vitro and in vivo experimental and clinical studies undertaken to investigate the mechanistic pathways by which these compounds exert their effects against the progression of CKD, particularly diabetic nephropathy and kidney fibrosis. The accumulated and collected data from preclinical and clinical studies revealed that these plants/bioactive compounds could activate autophagy, increase mitochondrial bioenergetics and prevent mitochondrial dysfunction, act as modulators of signaling pathways involved in inflammation, oxidative stress, and renal fibrosis. The main pathways targeted by these compounds include the canonical nuclear factor kappa B (NF-κB), canonical transforming growth factor-beta (TGF-β), autophagy, and Kelch-like ECH-associated protein 1 (Keap1)/nuclear factor erythroid factor 2-related factor 2 (Nrf2)/antioxidant response element (ARE). This review presented an updated overview of the potential benefits of these antioxidants and new strategies to treat or reduce CKD progression, although the limitations related to the traditional formulation, lack of standardization, side effects, and safety.

## 1. Introduction

Chronic kidney disease (CKD) is a significant public health concern, affects about 500 million people worldwide [1]. The pathogenesis of CKD includes several variables and steps, but the exact mechanism is still unknown. CKD pathogenesis’s leading and fundamental cause is linked to oxidative stress [2]. Fibrosis, tubulointerstitial inflammation, and glomerulosclerosis are structural changes seen in CKD patients’ glomeruli and tubular cells [3] The progression of CKD may advance to renal failure, and in that case, dialysis or kidney transplantation might be urgent and required [4].

Phytochemicals and other natural products are increasingly prevalent in promoting health and preventing various diseases, and they have been used in traditional and complementary medicine for many years [5]. Scientific researchers are looking for alternative treatments for CKD after seeing promising results from some clinical trials that used natural products or their isolated compounds [6,7]. The main potential therapeutic strategies in the management of CKD include modulation of the nuclear factor B (NF-κB) signaling pathway [8], activation of autophagy, prevention of mitochondrial dysfunction [9], activation of the nuclear factor erythroid 2-related factor 2 (Nrf-2) pathway, and inhibition of the transforming growth factor β (TGF-β) signaling pathway [10]. We searched scientific sources and article indexed databases, including PubMed and Google Scholar, by different keywords, including CKD, diabetic nephropathy; renal fibrosis; natural compounds; mechanistic pathways. This article reviews the most recent literature from animal models, in vitro, and clinical studies regarding the impact of antioxidants in the prevention and treatment of CKD. Although, natural antioxidant compounds have been shown to have protective benefits against CKD [11,12,13]. The molecular mechanisms by which these natural products and plant-derived compounds exerted their kidney-protective effects have yet to be identified. This article provides an overview of the role of different mechanistic pathways associated with CKD pathogenesis and the potential utility of targeting these pathways by natural antioxidants in the treatment of CKD. The selection criteria for these natural compounds were based on their favorable outcomes in preclinical and clinical research, as well as the fact that they were not covered in previous reviews, particularly the molecular pathways. This review identifies flaws in our present understanding of alternative therapies for chronic renal disease and areas where more research is needed. This is not a traditional literature review, but rather an eye-opening document meant to urge academics, particularly physicians, to conduct further research in this field.

## 2. Signaling Pathways That Predispose to the Progression of CKD

### 2.1. NF-κB Pathway

CKD is characterized by a state of systemic inflammation that contributes to CKD progression [14]. Multiple receptors are involved in precipitating chronic inflammatory renal injury, including Toll-like receptor 4 (TLR4) and tumor necrosis factor receptor 1 (TNFR-1) [15]. TLR4 is a pattern recognition receptor involved in the direct and indirect activation of the nuclear factor κB (NF-κB), the master regulator of inflammatory pathways [16]. It is pathologically activated in CKD through different ligands that are produced as a result of progressive renal tissue injury [17]. These ligands include high-mobility group box 1 (HMGB1), heat-shock proteins (HSPs), and components of the extracellular matrix [18,19]. Stimulation of TLR4 activates the adapter protein myeloid differentiation primary response 88 (MyD88), leading to the recruitment of interleukin-1 receptor-associated kinase 4 and 1 (IRAK 4/1) [20]. Consequently, IRAK 4/1 recruits TNF receptor-associated factor 6 (TRAF6), which in turn activates the NF-κB essential modulator (NEMO) complex, ultimately resulting in the nuclear translocation of NF-κB [21]. In the nucleus, NF-κB binds to specific 9–10 base pair, κB sites, thus activating the transcription of inflammatory mediators including TNF-α, interleukin 1β (IL-1β), interleukin 6 (IL-6), and monocyte chemoattractant protein 1 (MCP1) [22]. The inflammatory cytokines attract macrophages, which in turn secrete TNF resulting in the activation of TNFR-1 that recruits the scaffolds tumor necrosis factor receptor type 1-associated death domain (TRADD) and TNF receptor-associated factor 2/5 (TRAF 2/5) [23]. Therefore, TNFR-1 downstream signaling can activate apoptosis through recruitment of Fas-associated death domain (FADD) and activation of caspase-8, it can also induce the nuclear translocation of NF-κB through activation of the NEMO complex, further aggravating renal inflammatory injury [24].

### 2.2. Autophagy

One of the hallmarks of CKD is impaired autophagic flux, which is protective in renal disease [25]. Autophagy is activated in response to the accumulation of reactive oxygen species (ROS) and starvation [26]. Two main complexes are involved in the initiation of autophagy, class 3 phosphoinositide 3-kinase (PI3K) complex, which includes Beclin-1, and Unc-51-like kinase 1 (ULK-1) complex [27]. The class 1 PI3K suppresses ULK-1 by activating the mammalian rapamycin (mTOR) target, leading to a reduction in autophagic flux [28]. CKD is associated with an increase in the expression of microRNA-21 (MiR-21), which negatively regulates phosphatase and tensin homolog (PTEN) [29]. PTEN negatively regulates the class 1 PI3K; thus, the abundance of MiR-21 associated with CKD results in the inhibition of the ULK-1 complex and reduction in autophagic flux [30]. The second phase of autophagy involves the conversion of microtubule-associated proteins 1A/1B light chain 3B-I (LC3-I) to microtubule-associated proteins 1A/1B light chain 3B-II (LC3-II) to drive the elongation of autophagosome [31].

### 2.3. Mitochondrial Dysfunction

Mitochondrial dysfunction is a leading event contributing to CKD progression [32]. In diabetic nephropathy, hyperglycemia leads to an increase in the adenosine triphosphate (ATP) to adenosine monophosphate (AMP) ratio, a consequence of an elevation in nutrient availability that is associated with the diabetic milieu [33]. This increase in the level of ATP is attributed to an increase in oxidative phosphorylation, leading to a reduction in AMP and consequent inactivation of AMPK [34]. Similarly, excessive levels of circulating free fatty acids (FFA) activate renal CD36 leading to the inactivation of AMPK [35]. Hyperglycemia is also linked to a reduction in the NAD^+^ to NADH ratio due to increased electron transport chain activity related to the abundance of nutrients [36]. The reduced NAD^+^ levels lead to the inactivation of class III histone deacetylases the NAD^+^-dependent sirtuins [37]. These events coalesce to reduce the level of PGC1-α, the master regulator of mitochondrial biogenesis, leading to diminished mitochondrial biogenesis and mitochondrial dysfunction [38]. Dysfunctional mitochondria attract cytosolic dynamin-related protein1 (Drp1), which leads to excessive mitochondrial fission and mitochondrial fragmentation [39]. In addition, mitochondrial dysfunction leads to excessive fatty acid deposition due to the reduction in fatty acid oxidation [40]. Moreover, excessive ROS is released to the cytosol and mitochondrial apoptogenic factors leading to renal cell death [41].

### 2.4. Nrf-2 Signaling Pathway

The diabetic milieu is associated with the excessive activation of insulin and insulin-like growth factor-1 (IGF-1); this leads to phosphorylation of insulin receptor substrate 1 (IRS-1) [42]. Phosphorylated IRS-1 acts as an adapter protein that phosphorylates PI3K, which in turn converts phosphatidylinositol 4,5-bisphosphate (PIP2) to phosphatidylinositol 3,4,5-triphosphate (PIP3) [43]. PIP3 exposes a pleckstrin-homology domain that recruits the inactive protein kinase B (thereafter referred to as Akt), which is then activated and leads to the inhibition of glycogen synthase kinase 3β (GSK-3β), an enzyme involved in apoptosis [44]. One of the effects of active GSK-3β is the inhibition of the nuclear translocation of the nuclear factor erythroid 2-related factor 2 (Nrf-2); thus, its inhibition by Akt results in an increase in Nrf-2 dissociation from Kelch-like ECH-associated protein 1 (Keap-1) that is holding Nrf-2 in an inactive state in the cytosol [45]. The reduced levels of AMPK contribute to an increase in Keap-1-Nrf-2 complex and a reduction in binding of Nrf-2 to the antioxidant response element and transcription of antioxidant enzymes, including heme oxygenase-1 (HO-1), super oxide dismutase (SOD), catalase (CAT), and glutathione peroxidase (GPx) [46]. In addition, the inflammatory milieu related to CKD leads to reduced Nrf-2 transcriptional activity and renal antioxidant capacity [47].

### 2.5. TGF-β Signaling Pathway

Renal fibrosis is the last stage of CKD, characterized by tubulointerstitial fibrosis and glomerulosclerosis [48]. Transforming growth factor-β (TGF-β) is a critical mediator of renal fibrosis [49]. In a variety of cell types, activated TGF-β binds to transforming growth factor-beta receptor (TGF-β R), triggering an intracellular phosphorylation cascade involving the transcription factors mothers against decapentaplegic homolog 2 (SMAD2) and mothers against decapentaplegic homolog 3 (SMAD3) [50]. SMAD 2/3 complex with the common SMAD (SMAD4) translocates into the nucleus, leading to pro-fibrotic genes including collagen 1 and fibronectin that make up the extracellular matrix, proteins that are involved in epithelial to mesenchymal transition, and pro-fibrotic miRNA [51]. SMAD7, the inhibitory regulator in the TGF-β/SMAD signaling pathway, inhibits the activation of SMAD 2/3 via its negative feedback mechanism [52]. SMAD 7 is negatively regulated by microRNA-21 (miR-21); therefore, SMAD7 could be a therapeutic approach for CKD treatment [53]. The advanced glycation end products (AGEs) and angiotensin II (ANG 2) drive the activation of mitogen-activated protein kinase (MAPKs) that activate the TGF-β/SMAD signaling pathway [54,55]. Nitric oxide (NO) is also involved in inhibiting SMAD 2/3 and is therefore used as a therapeutic target for treating fibrotic kidney diseases [56].

## 3. Role of Antioxidants in the Prevention of CKD

The critical therapies for CKD patients depend on which stage they are in. To treat symptoms of CKD, pharmacological therapy, changes in diet and lifestyle, dialysis, and kidney transplantation are used. In addition to traditional therapy, scientists concentrate on the recent use of dietary supplements and novel natural products or their derivatives to reduce the high risk of CKD and limit the severity of this disease. In this regard, numerous studies and reviews have documented the potential impact of compounds with anti-inflammatory and antioxidant activities in CKD treatment [12,13,57,58]. Other studies have indicated that dietary interventions are essential in lowering inflammation and oxidative stress in CKD patients [57].

### 3.1. Medicinal Plants and Natural Compounds against CKD

Some plant extracts have been investigated previously in the treatment of CKD due to their possible therapeutic properties [58,59,60]. In this regard, recent experimental studies investigated the effects of *Phylanthus niruri* leaves aqueous extract (PN) on renal functions, structural alteration, and biomarkers of oxidative stress, inflammation, fibrosis, apoptosis, and proliferation in the diabetes mellitus (DM) rat model. The data indicated that PN could maintain normal kidney function and amended histopathological changes by improving oxidative stress markers such as thiobarbituric acid reactive substances (TBARS), superoxide dismutase (SOD), catalase (CAT) and glutathione peroxidase (GPx), inflammatory markers (NFkβ-p65, Ikk-β, TNF-α, IL-1β, and IL-6), apoptosis markers (caspase-3, caspase-9, and Bax), fibrosis markers (TGF-β1, VEGF and FGF-1) and proliferative markers such as proliferating cell nuclear antigen (PCNA) and Ki-67 in diabetic nephropathy (DN) rat model [61]. The authors reside the therapeutic effects of PN extract to the occurrence of antioxidant and anti-inflammatory activities of specific bioactive compounds (palmitic acid and linoleic acid).

Renal tubulointerstitial fibrosis is the predominant common mechanism of progressive kidney injury, leading to end-stage renal disease (ESRD). Wu and colleagues (2018) demonstrated the in vivo and in vitro anti-fibrotic effects of total flavonoids (TFs) derived from leaves of *Carya Cathayensis* and explored the underlying mechanisms [62]. TFs of *Carya Cathayensis* have been found to reduce renal fibrosis through a signaling pathway miR-21/Smad7, indicating their therapeutic function as an anti-fibrotic candidate. Additionally, it has been stated in a comprehensive review that flowers of *Abelmoschus manihot* (Linnaeus) Medicus (Malvaceae; *Flos A. manihot*) prevented the progression of CKD [63]. Data from in vivo studies in animal models of rabbits with glomerulonephritis [64], DN [65,66], and adriamycin-induced nephropathy [67,68] have revealed that flavonoids of *Flos A. manihot* have renoprotective effects, which are shown by the ability to alleviate proteinuria, apoptosis of podocytes, glomerulosclerosis and mesangial proliferation via various mechanisms focused on inhibition of caspases, amelioration of oxidative stress, infiltration reduction, and suppression of the p38 MAPK and serine/threonine kinase (Akt) pathways, as well as TGF-β1 and TNF-α expression. It has been documented in patients with glomerular disease that *Flos A. Manihot* was superior to losartan in proteinuria reduction [69].

*Astragalus*, the dried root of *Astragalus membranaceus*, is one of the most commonly used herbs for the treatment of kidney diseases in traditional Chinese medicine. There have been observations of many biological activities of *Astragalus*, including immunomodulatory [70], antioxidant [71], anti-inflammatory [72], and kidney protection [73].

In an in vitro model of oxidative stress, Shahzad et al. (2016) examined the renoprotective effect of ethanol, methanol, and aqueous crude extracts of roots of *A. membranaceus* on human kidney proximal tubular epithelial cells. The protective effect of *A. membranaceus* on renal damage related to anti-apoptotic and anti-inflammatory mechanisms [74]. Furthermore, it has been shown that *A. membranaceus* is capable of improving ischemic microvasculature and attenuating interstitial fibrosis by increasing NO output via eNOS activation and ROS scavenging in obstructed rat kidneys [75]. *A. membranaceus* treatment has been shown to alleviate kidney dysfunction by ameliorating serum creatinine, uric acid, sodium, and potassium levels in aged rats [76]. This treatment also preserved stable levels of eGFR and postponed the entrance in renal replacement therapy in patients with progressive CKD stage 4 [77]. Meta-analysis of the beneficial effect and the clinical value of *A. membranaceus* has shown that *Astragalus* therapy prevents the progression of DN and improves renal functions (BUN, SCr, CCr, and urine protein) and serum albumin levels in patients with DN [78,79]. Previous reports have been shown that treatment with *Astragalus* reduces proteinuria and enhances hemoglobin and serum albumin [59].

Recently, there is a commonly used formula of *Astragalus mongholicus* Bunge and Panax notoginseng (Burkill) F.H. Chen formula (APF) in China clinics for CKD treatment. It has been reported that this formula inhibits renal inflammatory injuries in DN by upregulation autophagy via suppressing mTOR and activating PINK1/Parkin signaling pathways [80]. Furthermore, Zuo et al. demonstrated that *Astragalus mongholicus* attenuated renal fibrosis in a rat model of unilateral ureteral obstruction (UUO) through the reduction in TGF-β1 and α-smooth muscle actin (alpha-SMA) expression [81]. Treatment by *Astragalus mongholicus* also improved the histopathological alterations comparable to the established renoprotective drug losartan. The same author reported that *Astragalus mongholicus* suppressed the transdifferentiation of renal epithelial tubular myofibroblasts halting the progression of renal interstitial fibrosis in the same model [82]. In another model of CKD, *Astragalus mongholicus* in combination with *Angelica sinensis* reduced renal fibrosis in chronic puromycin aminonucleoside-induced nephrosis by attenuating the expression of TGF-β1 and impeding renal macrophages localization comparable to renin-angiotensin-aldosterone system (RAAS) blockade by enalapril [83]. A gene microarray study revealed that *Astragalus mongholicus* fed DN mice had altered gene expression related to metabolism, immunity, and inflammation that positively impacted the disease state [84].

In eastern Asian countries, the azuki bean (*Vigna angularis*) is widely farmed and is considered one of the essential crops. The major constituents of *Vigna angularis* are the polyphenolic proanthocyanidins that are characterized by their ability to scavenge ROS [85]. In a recently published study, *Vigna angularis* administration mitigated kidney injury by increasing the expression of glutathione (GSH) and light chain 3B II (LC3B-II), as well as reducing the expression of heme oxygenase-1 (HO-1), p47phox (NADPH oxidase subunit), and p62/sequestosome 1 (p62) in STZ-induced DN rats [86]. An earlier study using the same model of DN demonstrated that treatment by seeds coats of *Vigna angularis* attenuated oxidative stress damage by reducing MDA, reduced inflammation as portrayed by decreased infiltration of macrophage, and downregulation MCP-1 gene expression [87]. The aqueous extract of *Vigna angularis* improved kidney function parameters in an experimental model of moderate chronic kidney disease [58].

In addition to medicinal plants, bee products have been used to supplement pharmacological compounds for their beneficial therapeutic activities. These effects include anti-inflammatory [88], antioxidants [89] and immunomodulatory activities [90]. A variety of preparations are produced by honeybees, such as royal jelly, propolis, bee wax, pollen grain, and bee venom [91]. A multitude number of bioactive compounds derived from honeybee products can be of value in the treatment of CKD and management of CKD complications [92,93].

Honeybee propolis is one of the significant beneficial components derived from honeybee products. Propolis contains bioactive compounds, including flavonoids such as chrysin and rutin, and phenolic compounds, such as caffeic acid and a stilbene derivative resveratrol [94]. The antioxidants characteristics of propolis are based on its content of flavonoids and phenolic compounds [95,96,97]. In a recent study, propolis was able to attenuate tubulointerstitial fibrosis by modulating canonical SMAD signaling pathway and JNK/ERK activation in the TGF-β cascade in a murine model of aristolochic acids-induced nephropathy [93]. Teles et al. demonstrated that Brazilian red propolis reduced hypertension and renal morphological alterations manifested by decreased serum creatinine, proteinuria, and infiltration of macrophages in 5/6 nephrectomized rats [98]. The authors suggested that the beneficial effects of Brazilian red propolis are due to its anti-inflammatory and antioxidant activity. Additionally, through reducing oxidative stress and blood pressure, Indonesian propolis extract has been shown to attenuate UUO-induced renal damage [99], and Iranian propolis extract could increase the antioxidant levels and amend histopathological alterations in the DN rats model [100]. In a randomized, double-blinded, and placebo-controlled clinical trial in humans, Brazilian green propolis significantly attenuated proteinuria in diabetic and non-diabetic CKD patients [92] and reduced inflammation in patients on hemodialysis [101].

Chrysin derived from bee propolis reduced kidney fibrosis induced by the accumulation of AGEs in in vitro and in vivo studies. In the in vitro study, chrysin treatment reduced AGEs-induced deposition of collagen, induction of α-SMA, and matrix metalloproteinases in human mesangial cells through downregulation of TGFβ1 and SMAD 2/3. Moreover, these findings were confirmed in an animal model of diabetic kidney disease [102]. Furthermore, chrysin attenuated adenine-induced CKD in rats through the reduction in inflammatory cytokines, boosting antioxidant status, and improving renal histopathological alterations [103]. Caffeic acid phenethyl ester (CAPE) is one of the constituents of honeybee propolis that has been shown to protect against lithium-induced renal tubular damage and oxidative stress in a rat model by boosting the antioxidant enzymes activities (SOD, CAT, GSH-Px) in renal tissue [104]. Caffeic acid also had anti-inflammatory effects in the model of diabetic nephropathy (DN) mice by reducing renal IL-6, IL-1β, TNF-α, and MCP-1 levels [105]. Pinocembrin, isolated from Mexican brown propolis, has been shown in the DN rat model to be able to improve lipid profile, glomerular filtration rate, urinary protein, prevent urinary biomarker increases, oxidative stress, and glomerular basement membrane thickness [106]. Bee venom is a natural toxin produced by honeybees and possesses a multitude of beneficial health activities [107]. Bee venom treatment attenuated renal fibrosis in unilateral ureteral obstruction (UUO)-induced CKD through a reduction in the expression of inflammatory markers (TNF-α and IL-1β), fibrotic markers (TGF-β1 and fibronectin), and α-SMA [108]. The mechanistic pathways targeted by some natural products against CKD are summarized in Table 1.

### 3.2. Small Bioactive Compounds against CKD

Tiny molecular compounds derived from natural products have shown potential therapeutic effects on CKD, as indicated in previous reports [109,110,111,112]. These compounds have antioxidant [113], anti-inflammatory [114], and immunomodulatory [115] activities that are linked to their disease alleviative property. Generally, bioactive compounds exert their antioxidant activity through direct ROS scavenging and indirectly by upregulation of the body’s antioxidant status. Moreover, these compounds exhibit anti-inflammatory activity through blunting inflammatory signaling cascade at the receptor and nuclear level, consequently attenuating the transcription of proinflammatory cytokines. The origin of bioactive compounds includes plants, animals, and biosynthetic sources.

#### 3.2.1. Berberine

Berberine is an isoquinoline alkaloid that occurs in different plants such as *Berberis vulgaris*, *Berberis aristata* and *Coptis chinensis*. It possesses several pharmacological properties that include anticancer [116], antibacterial [117], anti-hyperglycemic [118], cardioprotective [119], neuroprotective [120], antiatherosclerotic [121], and anti-inflammatory activities [122].

The therapeutic and protective effects of berberine are attributed to its antioxidant and anti-inflammatory activities. The molecular basis of the aforementioned effects is due to the ability of berberine to induce the expression of Nrf2 through the activation of AMPK, P38, and PI3K/Akt signaling pathways. Consequently, boosting the antioxidant defenses manifested by the increase in SOD, GSH, and HO-1 levels [123]. Additionally, through its direct inhibitory effect on IKK-β, berberine inhibits the activation and phosphorylation of IκB-α and, in turn, reduces the nuclear translocation of NF-κB [124]. Moreover, berberine could downregulate MAPK signaling leading to direct inhibition of release of proinflammatory cytokines [125].

**Figure 1:**
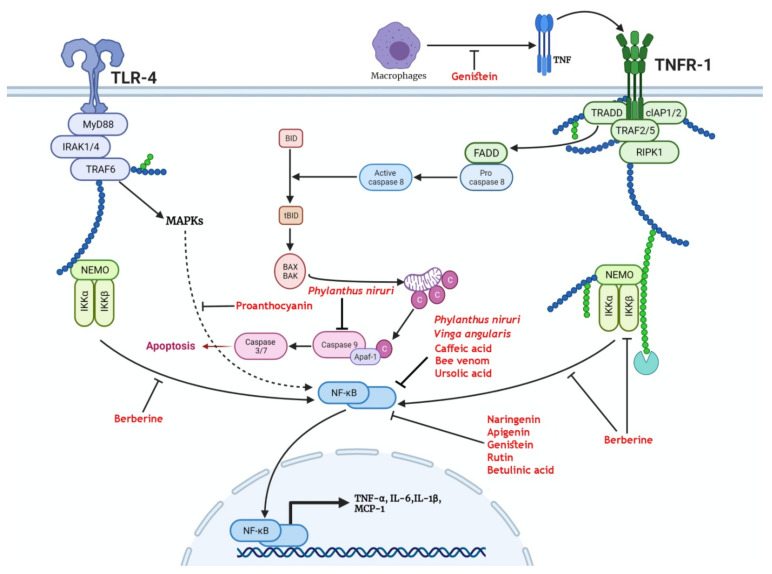
Medicinal plants and bioactive compounds that exert a protective effect through modulation of the nuclear factor kappa B(NF-κB) pathway. Activation of either the tumor necrosis factor receptor 1 (TNFR-1) by tumor necrosis factor (TNF) or Toll-like receptor 4 (TLR-4) by pathogen-associated molecular patterns (PAMPS) leads to the recruitment of IkappaB kinase α (IKKα) and IkappaB kinase β (IKKβ). These enzymes free NF-κB from its sequestration in the cytosol. The tumor necrosis factor receptor type 1-associated death domain (TRADD) scaffold recruits Fas-associated death domain (FADD) that activates caspase 8, leading to the truncation of BH3-interacting domain (BID) to form tBID. Truncated BID (tBID) activates BCL2-associated X protein (BAX) and BCL2-antagonist/killer 1 (BAK) mitochondrial translocation and release of cytochrome c that activates effector caspases. Genistein exerts a protective effect through the inhibition of activation of TNFR-1. Berberine inhibits the recruitment of the NF-κB essential modulator (NEMO) complex to TNFR-1 and reduces the activation of NF-κB. Proanthocyanins inhibit mitogen-activated protein kinases (MAPKs)-mediated activation of NF-κB. *Phylanthus niruri*, *Vinga angularis*, caffeic acid, bee venom, ursolic acid, naringenin, apigenin, genistein, rutin, and betulinic acid reduce the activation and nuclear translocation of NF-κB. As a consequence, the transcription and release of inflammatory cytokines and chemokines such as tumor necrosis factor-α (TNF-α), interleukin-6 (IL-6), interleukin-1β(IL-1β), and monocyte chemoattractant protein-1 (MCP-1) are reduced.

Berberine reduced the expression of NF-κB (Figure 1) in cultured mouse podocytes genetically modified to overexpress tumor necrosis factor (TNF) receptor-associated factor 5 (TRAF5), mimicking the raised levels of TRAF5 in CKD patients. Treatment by berberine reversed the reduced viability and increased apoptosis that was accompanied by the overexpression of TRAF5. The protective effect of berberine in this study is attributed to blunting inflammatory response that is associated with the upregulation of TRAF5 in CKD patients [125].

In a rat model of STZ-induced diabetic kidney disease, Zhu and colleagues reported that berberine reduced renal injury via the reduction in TLR4-dependent NF-κB-mediated inflammation [126]. In another recent study, the administration of berberine to diabetic mice attenuated the deranged lipid metabolism and mitochondrial bioenergetics associated with diabetic kidney disease. In this study, berberine induced the expression of PGC-1α, leading to enhanced mitochondrial function, reduced mitochondrial ROS formation, and diminished lipid deposition. Furthermore, these effects reduced injury of podocytes and increased the expression of nephrin and podocin, ameliorating diabetic-induced glomerulosclerosis and maintaining renal function [127]. Mitochondrial dynamics were also modulated by berberine in an in vitro and in vivo experiment of diabetic kidney disease [128]. Berberine treatment has been shown to reduce the expression of dynamin-related protein 1 (Drp1) and consequently reduce the mitochondrial fission induced by palmitate treatment in cultured mouse podocytes and diabetic mice. The mitochondrial modulatory effects of berberine reduced the apoptosis of podocytes, mitochondrial ROS generation, and fragmentation. Accordingly, berberine improved the changes in podocytes’ structure associated with the diabetic milieu and mesangial cells along with stiffness of glomeruli [128]. Berberine therapy could improve diabetic kidney disease in patients with type 2 diabetes, as shown by the decreased level of the urinary albumin/creatine ratio (UACR) and serum cystatin C (Cys C) [129].

#### 3.2.2. Ursolic Acid

Ursolic acid is a pentacyclic triterpenoid naturally present in leaves of various plants (rosemary, marjoram, lavender, thyme, and organum), fruits (apple fruit peel), flowers, and berries [130,131]. Ursolic acid in animal models and in vitro studies showed a protective effect against renal damage [132,133,134]. Additionally, it has been documented that ursolic acid has many beneficial effects, including anti-inflammatory [135], antioxidant [136], and antitumor activities [137], and prevent the progressing of chronic diseases through modulating several signaling pathways [138,139].

**Figure 2:**
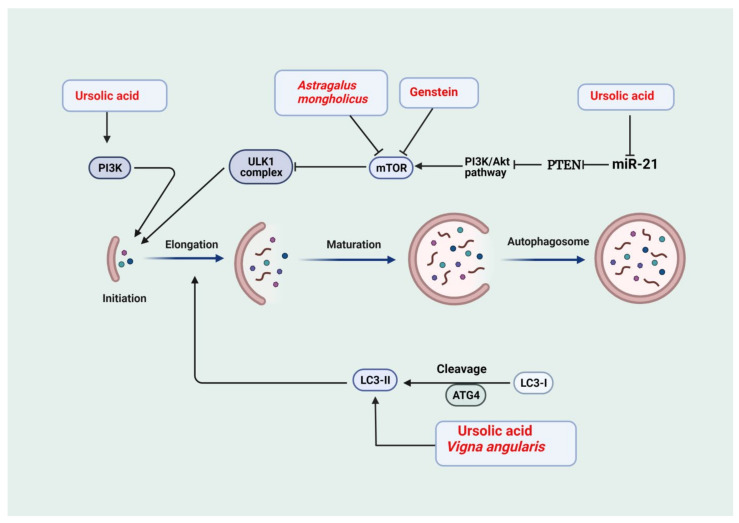
Medicinal plants and bioactive compounds that activate autophagy. The phosphatidylinositol-3-kinase (PI3K)/Akt and the mammalian target of rapamycin (mTOR) (PI3K/Akt/mTOR) pathway is a negative modulator of the Unc-51-like kinase 1 (ULK-1) complex. Activation of phosphoinositide 3-kinase (PI3K) and ULK-1 complex is required for initiation. Light chain 3-I (LC3-I) is cleaved to light chain3-II (LC3-II) to drive the elongation phase. This is followed by maturation to form the autophagosomes. Phosphatase and tensin homolog (PTEN) is a negative modulator of the PI3K/Akt/mTOR pathway, and microRNA-21 (miR-21) inhibits PTEN. Ursolic acid activates autophagy through inhibition of miR-21, thus allowing PTEN to inhibit PI3K/Akt/mTOR pathway, leading to activation of the ULK-1 complex. In addition, ursolic acid activates the PI3K complex. Therefore, ursolic acid is involved in the initiation phase of autophagy. *Astragalus mongholicus* and genistein inhibit mTOR, leading to activation of ULK-1. Ursolic acid and *Vinga angularis* drive the formation of LC3-II and the elongation phase.

In the rat model of adenine-induced chronic tubular injury, administration of ursolic acid (30 mg/kg Body weight) for 28 days inhibited the activation of TGF-β/Smad signaling and in turn reduced extracellular matrix (ECM) proteins such as fibronectin (FN) and collagen, suppressing the development and progression of renal fibrosis [140]. Additionally, in another study, ursolic acid impeded the progression of tubulointerstitial fibrosis through attenuating epithelial to mesenchymal transition in renal tubular epithelial cells along with a reduction in markers of fibrosis such as collagen 1, FN, α-SMA, and increase in E-cadherin in a UUO mouse model of CKD. Furthermore, treatment by ursolic acid reduced the expression of α-SMA, snail1, slug, TGF-β1, and p-smad3 and prevented the loss of E-cadherin in an in vitro model of TGF-β1-treated HK-2 cells [141]. Ursolic acid significantly increased protein synthesis and reduced the breakdown of proteins in vivo and in vitro models of CKD-induced muscle wasting. These effects are attributed to the ability of ursolic acid to downregulate the expression of myostatin and inflammatory cytokines such as TGF-β, IL-6, and TNFα [142]. In addition, ursolic acid treatment for 10 weeks alleviated diabetic kidney injury by regulating the angiotensin II type 1 receptor-associated protein (ARAP1)/angiotensin II type 1 receptor (AT1R) signaling pathway and reducing the expression of NADPH oxidase 2 (NOX2), NADPH oxidase 4 (NOX4), 8-hydroxydeoxyguanosine (8-OHdG), TGF-β1, FN, Col IV, IL-1β and IL-18 at mRNA and protein level leading to inhibition of ECM accumulation, fibrosis, oxidative stress and renal inflammation [143]. The ursolic acid treatment induces the antioxidant defenses in renal tissue as exemplified by increased GSH levels and CAT, SOD, and GSH-Px enzyme activities in diabetic rats. Moreover, treatment by this bioactive compound reduced oxidative stress as shown by decreased tissue lipid peroxidation [144]. Interestingly, ursolic acid alleviated renal injury in the alloxan-induced diabetes model through the reduction in proinflammatory cytokines [145]. Uncontrolled hyperglycemia is implicated in impaired autophagy in renal cells; consequently, targeting the impaired autophagic repair machinery has the potential to amend diabetic renal injury [146]. In this regard, cultured murine podocytes exposed to high glucose levels showed reduced markers of autophagy such as LC3II and Beclin1 that were reversed upon ursolic acid treatment [147] (Figure 2). Consequently, relieving hyperglycemia-induced injury to podocytes as manifested by restoration of synaptopodin, podocin, and nephrin levels. The renal protective effects of ursolic acid were related to its inhibitory action on the PI3K/Akt/mTOR pathway via an enhancement in the expression of PTEN secondary to downregulated miR-21. The protective effect of ursolic acid was abrogated following treatment by autophagy inhibitor 3-MA further confirming its protective action through modulation of impaired autophagy [147].

#### 3.2.3. Naringenin

Naringenin is one of the essential naturally occurring flavonoids found mainly in certain eatable fruits, such as tomatoes and citrus species [148]. It exhibits limited solubility in water, whereas its solubility is higher in alcohol. It is derived from the hydrolysis of naringin or narirutin [149]. The natural biosynthesis of naringenin is a multi-step process that begins with converting phenylalanine to cinnamic acid viz the action of phenylalanine ammonia-lyase. Subsequently, several enzymes catalyze multiple reactions, ultimately leading to the formation of naringenin [150].

This reaction yields a meager amount of the bioactive compound; therefore, several attempts were carried out to increase the yield of naringenin through genetic engineering. The highest yield of naringenin was obtained from genetic manipulation of Escherichia coli [151]. Following oral ingestion, naringenin is extensively degraded by the gut microbiota yielding a wide array of breakdown products, leading to reduced bioavailability [152]. Interestingly, the bioavailability of naringenin was found to be lower in trained athletes [153].

Naringenin revealed many pharmacological activities (for example but not limited to) on human health, which includes antidiabetic properties [154], antimetastatic [155], neuroprotection [156], suppression of proliferation of endometriosis cell lines, and increasing their apoptosis [157], as well as reducing incidence of cardiovascular diseases [158]. Generally, like most flavonoids, naringenin exerts beneficial effects owing to its antioxidant property. The antioxidant activity of naringenin is attributed to its modulatory effect on Nrf2, a major transcriptional factor for cellular antioxidant defenses [159,160]. Furthermore, naringenin possesses anti-inflammatory properties mainly through its inhibitory activity on NF-κB and subsequent reduction in transcription of proinflammatory cytokines [161,162]. Perturbations in TGF-β signaling lead to the upregulation of pro-fibrotic transcriptional mediators that play a role in CKD progression [163].

In this context, naringenin treatment in mice with obstructive nephropathy attenuated renal fibrosis by inhibiting the expression and activation of smad3, a crucial pro-fibrotic factor, leading to a reduction in collagen I and α-SMA in renal tissue. Similar findings were obtained from cultured normal rat kidney tubular epithelial cell line (NRK52E) subjected to recombinant human TGF-β1 [164].

One of the etiologies of CKD is hypercholesterolemia through induction of inflammatory renal damage and oxidative stress [165]. Moreover, increased plasma cholesterol levels interfere with purinergic signaling in platelets promoting their aggregation [166]. In a rat model of high cholesterol diet-induced renal failure, naringenin treatment attenuated the hydrolysis of adenine nucleotides that is stimulated by high plasma cholesterol levels. As a consequence, several indices of oxidative stress and inflammation were improved by naringenin, including inducible NO synthase (iNOS), TNF-α, IL-6, and NF-κB. Moreover, naringenin reversed the reduction in ectonucleoside triphosphate phosphohydrolase (NTPDases) and ecto-5’-nucleotidase (CD73) activities that are associated with inflammatory signaling and enhanced platelets aggregation [167]. Naringenin has a potential renoprotective effect in diabetes, which is indicated by significant amelioration of diabetic kidney impairment. In this regard, naringenin-treated diabetic rats showed reduced renal oxidative stress, inflammation, and apoptosis compared to untreated diabetic rats. In this study, some parameters were improved by naringenin, including MDA, SOD, CAT, GSH, as well as TGF-β1 and IL-1 [168]. Additionally, Ding and colleagues investigated the potential role of naringenin in ameliorating diabetic kidney disease through modulation of peroxisome proliferators-activated receptors (PPARs) with subsequent normalization of CYP4A expression concomitant increase in 20-hydroxyeicosatetraenoic acid (20-HETE) in diabetic mice. Similarly, the level of PPARs-CYP4A-20-HETE was recovered by naringenin in NRK-52E cells exposed to high glucose [169].

#### 3.2.4. Apigenin

One of the significant flavonoids occurring in plants is flavone apigenin. It is widespread in the family *Asteraceae,* for example, the genera *Tanacetum* [170], Matricaria [171], Achillea [172], and Artemisia [173]. Apigenin is present in different forms in the family of Lamiaceae, such as the aglycone, O-glucosides, glucuronides, C-glucosides, acetylated derivatives, and O-methyl ethers [174].

Apigenin is formed from phenylalanine identical to naringenin through the phenylpropanoid pathway. Phenylalanine is converted to cinnamic acid and then to p-coumaric acid that is activated by CoA followed by condensation with three malonyl-CoA residues and aromatization to form chalcone. Chalcone isomerization yields naringenin oxidized by a flavanone synthase to apigenin [175]. Like naringenin, apigenin possesses antioxidant and anti-inflammatory activities that can benefit the prevention of the progression of chronic kidney disease. A previous review article highlighted the therapeutic potential of apigenin in several diseases with [174].

Mechanistically, apigenin exerts its therapeutic effects by modulating multiple functions such as induction of cell cycle arrest and apoptosis in cancer cells [176]. Apigenin modulates the cell cycle by altering the expression of cyclin-dependent kinases (CDKs). Moreover, apigenin induces apoptotic cell death through mitochondrial (intrinsic) and non-mitochondrial (extrinsic) pathways. In the intrinsic pathway, apigenin acts directly on the mitochondrial membrane, leading to the depolarization of the mitochondrial membrane potential and causing an increase in the mitochondrial permeability transition, ultimately stimulating the release of cytochrome C and subsequently activating the cytoplasmic executioner caspases [177]. Apart from its mitochondrial cell death-promoting activity, it upregulates the expression of death receptor 5 (DR5), inducing tumor necrosis factor-related apoptosis-inducing ligand (TRAIL)-mediated caspase eight activations [178]. The anti-inflammatory activity of apigenin is related to its modulation of several pathways such as PI3K/Akt, p38/MAPK, stabilization of IKB leading to a reduction in the nuclear translocation of NF-κB, and inhibition of COX-2 [179]. Apigenin promotes the cellular antioxidant capabilities by enhancing the expression of antioxidant enzymes (SOD, CAT, and GSH-synthase) and induction of Nrf-2 nuclear translocation [180,181].

**Figure 3:**
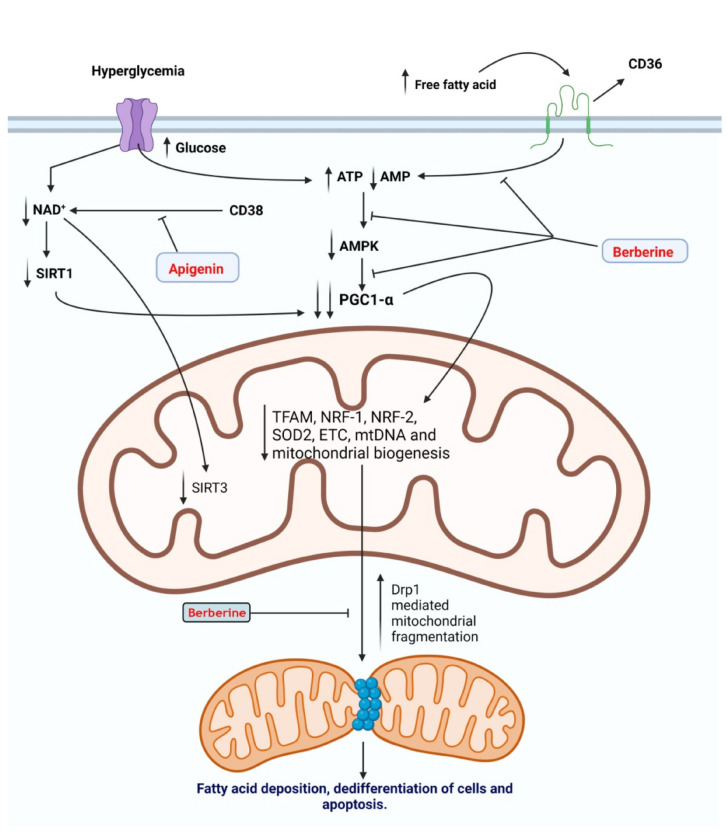
Medicinal plants and bioactive compounds that increase mitochondrial bioenergetics and prevent mitochondrial dysfunction. Hyperglycemia is accompanied by an increase in oxidative phosphorylation, leading to an increase in the adenosine triphosphate (ATP) to adenosine monophosphate (AMP) ratio and a reduction in nicotinamide adenine dinucleotide (NAD^+^): nicotinamide adenine dinucleotide hydrogen (NADH^+^) ratio. Reduction in AMP and NAD leads to inactivation of activated protein kinase (AMPK) and sirtuin-1 (SIRT1), respectively. This, in turn, leads to the inhibition of peroxisome proliferator-activated receptor-gamma coactivator -1α (PGC1-α), the master regulator of mitochondrial biogenesisSimilarly, an increase in free fatty acids (FFA) levels activates a cluster of differentiation 36 (CD36), leading to the inactivation of AMPK and PGC1-α. Reduced mitochondrial biogenesis leads to recruitment of cytosolic Drp1 to the outer mitochondrial membrane and an increase in mitochondrial fission. Consequently, mitochondrial fragmentation leads to a reduction in fatty acid oxidation and accumulation of FFA, leading to dedifferentiation of renal cells and apoptosis. Berberine inhibits the inactivation of AMPK and the recruitment of dynamin-related protein1 (Drp1) to the mitochondrial membrane. Thus, maintaining mitochondrial biogenesis and bioenergetics and preventing mitochondrial dysfunction that results from hyperglycemia and the increase in levels of FFA. Apigenin inhibits CD38-mediated consumption of NAD, thus preventing the inactivation of sirtuin-1 (SIRT1) and sirtuin-3 (SIRT3).

Previous studies have shown the renoprotective effect of apigenin through the attenuation of renal fibrosis via activation of AMPK leading to the inactivation of the TGF-β1/Smad2/3 signaling pathway [182,183]. Uremia associated with the build-up of toxic compounds secondary to renal dysfunction is a major contributor to endothelial dysfunction and cardiovascular disease. In an in vitro study, cultured human endothelial cells recovered from umbilical veins were subjected to uremic sera isolated from ESRD patients on peritoneal dialysis. Concurrent treatment by apigenin attenuated the dysfunction associated with the exposure of endothelial cells to uremic sera. The authors concluded that apigenin exerted its beneficial effect by reducing oxidative stress and inhibiting p38/MAPK activation [184]. The NAD+-dependent Sirt3 is critical for the proper functioning of mitochondrial respiration and redox state in the high energy demanding renal tubular epithelial cells [185]. Depletion of NAD+ leads to inactivation of mitochondrial Sirt3 compromising mitochondrial function and leading to excessive ROS generation [186]. In a diabetic rat model, apigenin alleviated mitochondrial-induced oxidative stress due to reduced Sirt3 activity through inhibition of CD38-mediated consumption of NAD+ in renal tubular cells [187]. In another study, Li et al. demonstrated the ability of apigenin nanoparticles to reduce renal damage associated with diabetic kidney disease through activation of Nrf2/HO-1 and inhibition of the NF-κB signaling pathway [188] (Figure 3).

#### 3.2.5. Genistein

Genistein is a typical, naturally occurring soy isoflavone isolated from Genista tinctoria for the first time [189]. Due to the similarities in structure to estradiol, genistein possesses estrogen-like functions [190]. Genistein has rapid metabolism in the liver and the intestine after 30 min of administration [191]. As reported in a previous review, genistein has multiple significant impacts in the treatment of metabolic diseases such as obesity, diabetes, and atherosclerosis, as well as anti-proliferative activity against cancer [192]. It also can control, prevent and treat various body disorders by modulating specific molecular pathways [193,194,195].

Among these pathophysiological pathways, genistein has been shown to be capable of preventing diabetic osteoporosis through activation of β-catenin, runt-related transcription factor 2 (Runx-2), inhibition of receptor activator of nuclear factor κB ligand (RANKL) and peroxisome proliferator-activated receptor- γ (PPAR-γ) signaling pathways [196]. One of the possible mechanisms through which genistein induces apoptosis and cell cycle arrest in cancerous cells is by the inhibition of NF-κB and modulating the levels of anti-apoptotic protein Bcl-2 and proapoptotic protein (Bax), as well as modulating caspase-3 and p38/MAPK signaling pathways [197,198]. In addition, genistein was reported to improve glycated hemoglobin, blood glucose levels, triglyceride and decrease oxidative stress marker (MDA) in postmenopausal women with type 2 diabetes mellitus [199]. A substantial number of studies have reported the anti-inflammatory properties of genistein through inhibition of COX-2 and downregulation of proinflammatory markers such as TNF-α, IL-1β, IL-6, and NF-κB [200,201,202].

Concerning the anti-fibrotic effect of genistein, a study in a rat diabetic model revealed that genistein treatment attenuated renal fibrosis as shown by lower levels of TGF-β1, p-Smad3, and collagen IV compared to diabetic untreated rats [203]. Similar observations were drawn from an early study involving cultured rat mesangial cells exposed to high glucose [204]. It has been reported that renal autophagic repair is impaired in CKD [205]. This was confirmed in an in vitro study of cultured murine podocytes treated by high glucose that showed unsustained autophagic repair compared to cells treated by genistein. The authors stipulated that genistein enhances autophagy through inhibition of mTOR signaling. Additionally, the autophagic repair was sustained in the presence of chloroquine and siRNA targeting the protein MyD88 [206]. The antioxidant and anti-inflammatory properties of genistein were shown to protect against diabetic renal damage in a mouse model of alloxan-induced diabetes. The markers of inflammation, including Cox-2, MCP-1, TNF-α, and NF-κB, were reduced in the genistein-treated group compared to untreated diabetic mice. Similarly, genistein reduced the markers of oxidative stress (MDA) and increased the cellular antioxidant defenses (Nrf2, GPx, SOD, and HO-1) [207].

In an experimental study involving healthy subjects and hemodialysis patients, withdrawn whole blood and isolated mononuclear cells pre-incubated with genistein showed a lower activatory tendency and low levels of TNF-α secretion in whole blood and monocytes of hemodialysis patients exposed to LPS. However, no significant alteration in the level of IL-6 and IL-10 was found in the treated and untreated healthy and hemodialysis patient groups. The authors concluded that the anti-inflammatory activity of genistein was due to its inhibitory activity on TNF-α release from monocytes in response to proinflammatory stimuli [208].

#### 3.2.6. Rutin

Rutin is a glycoside of the flavonoid quercetin that can be naturally present in many plants, including citrus fruits, buckwheat, tobacco, tea, and passion flower [209,210]. Rutin nomenclature is derived from the plant Ruta graveolens and also can be called rutoside, quercetin-3-O-rutinoside, rutinum, sophorin, and vitamin P [211]. Rutin’s chemical structure consists of flavonolic aglycone quercetin plus rutinose disaccharide.

**Figure 4:**
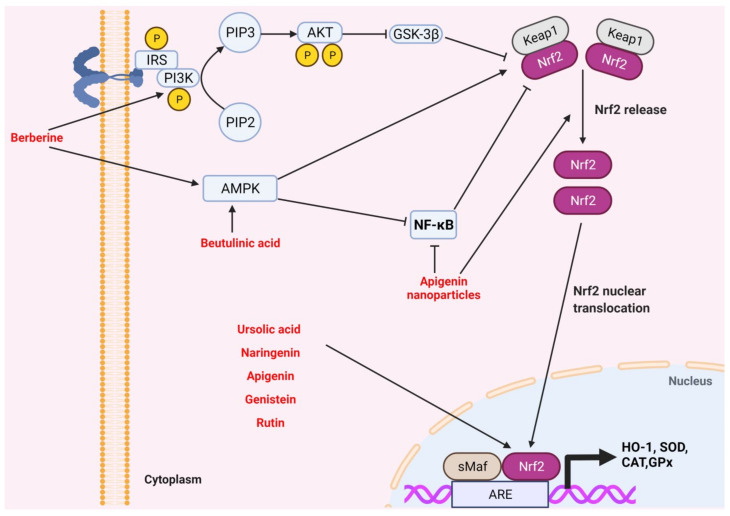
Medicinal plants and bioactive compounds that activate the Kelch-like ECH-associated protein 1/nuclear factor erythroid 2-related factor 2/antioxidant response element (Keap1/Nrf2/ARE pathway). Activation of the insulin receptor recruits PI3K that converts phosphatidylinositol 4,5-bisphosphate (PIP2) to phosphatidylinositol 3,4,5-triphosphate (PIP3). PIP3 activates Akt, which in turn inhibits glycogen synthase kinase 3β (GSK-3β), relieving its inhibitory action on cytosolic Nrf2. Activation of AMPK releases cytosolic Nrf2 from Keap1 in a direct and an indirect manner. The nuclear translocation of cytosolic Nrf2 leads to activation of the antioxidant response element and transcription of antioxidant enzymes such as heme oxygenase-1 (HO-1), super oxide dismutase (SOD), catalase (CAT), and glutathione peroxidase (GPx). Berberine directly activates PIP3, leading to AKT-mediated activation of cytosolic Nrf2 through inhibition of GSK-3β. In addition, berberine and betulinic acid activate AMPK, leading to the release of cytosolic Nrf2. Apigenin nanoparticles inhibit NF-κB, thus allowing for the release of Nrf2. Ursolic acid, naringenin, apigenin, genistein, and rutin increase the nuclear level of Nrf2 and its transcriptional activity, leading to an increase in the expression of antioxidant enzymes.

Pharmacological reports have proposed that rutin has antioxidant and anti-inflammatory effects, and therefore might be considered for the treatment and management of CKD. The antioxidant properties of rutin are owing to its ability to suppress the oxidation process, scavenge released free radicals and enhance antioxidant enzyme activities [212]. On this point, rutin treatment has shown strong DPPH radical scavenging activity and effective inhibition of lipid peroxidation [213]. Additionally, the antioxidant activity of rutin is based on promoting the release of antioxidant enzymes induced by Nrf2 upregulation (Figure 4) [214,215]. Rutin had anti-inflammatory properties primarily through inhibiting NF-κB pathway activation and subsequently reducing the release of proinflammatory cytokines such as TNF-α, IL-1β, and IL-6. In this respect, rutin has been shown to have anti-inflammatory effects in a mouse model of LPS-induced mastitis through inhibition of the NF-κB signaling pathway and attenuation of endoplasmic reticulum (ER) stress [216].

Animal model results showed that rutin treatment (100 mg/kg/day) for eight weeks improved renal structure (tubulointerstitial fibrosis) and function (urea, creatinine, uric acid, and proteinuria) in adenine-induced CKD by reducing the expression of biomarkers of oxidative stress (HO-1) and inflammation phospholipase A2 (PLA-2) [217]. In another study, rats that underwent 5/6 nephrectomy showed that rutin administration for 20 weeks decreased renal fibrosis and reduced proteinuria, the authors of the study related these effects of rutin to its antioxidant properties and the suppression of the TGFβ1-Smad signaling pathway [218]. Wang and colleagues made similar observations; they reported that rutin treatment attenuated interstitial renal fibrosis in UUO rats, which was shown by inhibiting extracellular matrix accumulation through reducing expression of type I/III collagen and fibronectin along with preventing the epithelial-mesenchymal transition in renal tubular cells by decreasing α-SMA expression and reserving E-cadherin expression. The authors correlated the renoprotective role of rutin with its anti-inflammatory effects and TGF-β1/Smad3 signaling pathway inhibition [219]. The renoprotective effect of rutin on high cholesterol diet (HCD)-mediated renal injury has also been observed when co-treated with ascorbic acid through inhibiting oxidative stress and boosting cellular antioxidant defenses [220].

It has been reported that rutin therapy markedly prevented hyperglycemia-induced endothelial barrier dysfunction in endothelial cells of human renal glomeruli exposed to hyperglycemia by inhibiting the signaling pathway of ras homolog gene family, member A/ Rho-associated protein kinase (RhoA/ROCK) by the activation of Nrf2 [221].

A study in a model of alloxan-induced diabetic kidney disease (DKD) in rats revealed that rutin in combination with ramipril alleviated renal damage through a reduction in oxidative stress and renal fibrosis; the authors reported reduced TGF- β1 and increased podocin levels in rats treated by the combination in comparison to untreated rats and rats treated with ramipril alone. Histopathological examination demonstrated improved tubulointerstitial architecture in rats treated by rutin in combination with ramipril. Moreover, the addition of rutin further attenuated endoplasmic reticulum stress in renal cells, as shown by reduced levels of unfolded protein response markers GRP78 and CHOP [222]. Another study in alloxan-treated rats showed that rutin possesses renoprotective activity as shown by its ability to reduce the biochemical markers of renal injury such as serum creatinine, blood urea nitrogen, and ketone bodies. Furthermore, rutin-treated rats had lower metabolic acidosis associated with diabetic kidney injury compared to untreated rats, as shown by reduction in gene expression of aquaporin-2 (AQP2), aquaporin-3 (AQP3), and the type 2 vasopressin receptor (V2R). The protective activity of rutin extended to the reduction in serum triglycerides and cholesterol and improvement in histopathological study results, particularly fibrosis and diabetic ketoacidosis [223].

Renal fibrosis and hyperglycemia-induced glycosylation of proteins and fats are associated with the progression of DKD. In this regard, the administration of rutin attenuated renal fibrosis by reducing the expression of collagen IV, laminin, TGF-β1, p-Smad 2/3, and connective tissue growth factor (CTGF) in a rat model of STZ-induced DKD. Moreover, rutin reduced the fasting blood glucose, serum creatinine, BUN and urinary protein level in diabetic rats compared to untreated rats. In addition, oxidative stress and AGEs were reduced in the rutin-treated group, whereas the anti-fibrotic protein p-smad 7 was increased. Electron microscopy examination revealed that rutin reduced mesangial cell expansion seen in the STZ-treated group and prevented reducing the thickness of the glomerular basement membrane [224].

#### 3.2.7. Proanthocyanin

Proanthocyanidins are naturally polyphenolic compounds in the majority of plants with very potent antioxidants properties. Chemically, they are produced from flavan-3-ols polymerization resulting in high molecular weight compounds called condensed tannins. They are considered oligomers or polymers of catechins or flavanols, joined by carbon-carbon bonds, with a complex chemical structure [225].

The predominant important properties of proanthocyanidins are their antioxidant activity by inhibiting the formation of free radicals in the body [226]. Scavenging these free radicals or preventing their production has beneficial effects and is considered the main strategy in treating various diseases. Proanthocyanidins also exhibit anti-inflammatory activity by inhibiting the activation of NF-κB signaling cascade via MAP kinases and consequently reducing the release of proinflammatory cytokines such as IL-1β, IL-6, and IL-8 [227]. Additionally, proanthocyanidins have been reported to have several biological functions, including antidiabetic, anti-atherosclerotic, and cardioprotective activities [228,229,230], as well as cardiovascular diseases control and treatment [231].

In an experimental animal model of mice undergoing unilateral I/R, administration of grape seed proanthocyanidins extract (GSPE) attenuated chronic renal fibrosis and reduced inflammation. In addition, GSPE improved parameters of renal function such as BUN and creatinine and reduced pathological alterations and inflammation. The authors attributed the renoprotective effect of GSPE to the inhibition of high-mobility group box 1 (HMGB1) production [232]. In another study, GSPE administration (125, 250, or 500 mg/kg/day orally) for eight days alleviated chronic renal fibrosis and inflammation in UUO mice by inhibiting the complement component 3 (C3) produced by macrophages. This finding was supported in an in vitro experiment of cultured primary renal tubular epithelial cells (PTEC) in which treatment with GSPE inhibited C3 and decreased HMGB1 through suppressing the HMGB1/TLR4/p65/TGF-β1 signaling pathway. Furthermore, GSPE inhibited myofibroblast activation through TGF-β1/Smad2/3 signaling pathways in normal rat kidney fibroblast (NRK-49F) cells [233].

Many studies have been performed to examine the beneficial effect of treatment with GSPE on the prevention of diabetic kidney disease progression. In this respect, it has been reported that treatment with GSPE (500 mg/kg/day) for 24 weeks attenuated diabetic nephropathy by suppressing AGEs/RAGE axis and downregulation in the expression of connective tissue growth factor (CTGF) at mRNA and protein levels [234]. In another study, STZ-induced diabetic rat model, co-treatment of proanthocyanidin with thymoquinone exerted renoprotective effect as shown by a decrease in the elevated levels of urea, creatinine, nitric oxide, MDA, and IL-6 in addition to an increase in antioxidant enzymes (GSH and SOD) compared with untreated diabetic rats [235]. Similarly, GSPE treatment slowed the progression of DKD through the activation of the Nrf2 signaling pathway [236]. Additionally, in a rat model of nephropathy associated with type 2 diabetes induced by STZ and a high-fat diet, Boa et al. showed that GSPE treatment for 16 weeks could alleviate renal injury by inhibiting inflammation and boosting antioxidant activity [237]. GSPE also protected the kidneys in STZ-induced diabetic rats by attenuating programmed cell death induced by endoplasmic reticulum stress via reducing the expression of glucose-regulated protein levels 78 (GRP78), p-ERK, and caspase 12 levels [238].

In a recent review study, the utility of food containing proanthocyanidins for middle-aged and older women was documented. Proanthocyanidins have been documented to contribute to preventing a wide range of diseases, as well as improving menopausal disorders, kidney function, and skin injury [239]. Furthermore, intake of proanthocyanidins has been reported to enhance renal function in women over 75 years of age [240]. As endothelial dysfunction is associated with human diabetic nephropathy, a randomized, double-blind, placebo-controlled study has revealed that GSPE intake for 12 weeks maintains vascular elasticity and normal blood pressure in volunteers with prehypertension [241].

#### 3.2.8. Betulinic Acid

Betulinic acid is a pentacyclic lupane-type triterpenoid that occurs naturally in many plants, particularly birch trees [242]. Previous reports have shown that betulinic acid possesses comprehensive biological activities including anti-viral [243], antioxidant [244], hepatoprotective [245], renoprotective [246] and anti-inflammatory [247]. Furthermore, betulinic acid has anti-proliferative and anti-apoptotic properties in many cancer cell types [248,249]. In a recently published review, the molecular mechanisms underlying the antitumor activity of betulinic acid were summarized [250]. Two main steps are involved in the betulinic acid biosynthesis process: lupeol synthesis by 2.3-oxidosqualene cycling and subsequently oxidation by cytochrome P450 enzymes in the C28 position (CYP) [251].

**Figure 5:**
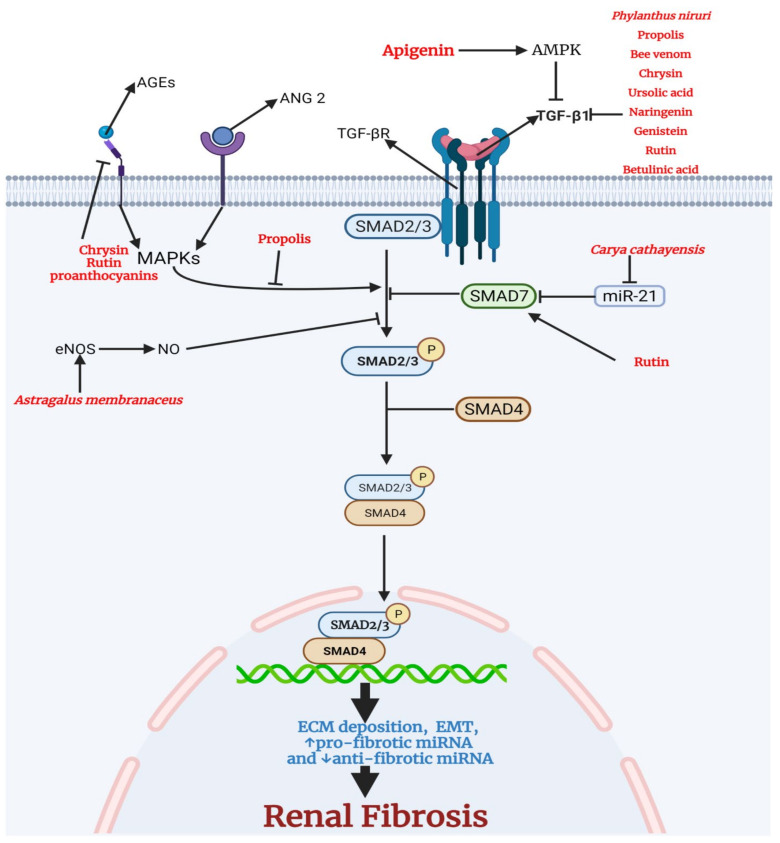
Medicinal plants and bioactive compounds with anti-fibrotic activity. The canonical transforming growth factor-beta1 (TGFβ1) pathway involves the binding of TGFβ1 to transforming growth factor-beta receptor (TGFBR). This leads to the phosphorylation and activation of small mothers against decapentaplegic 2/3 (SMAD 2/3). SMAD7, the inhibitory SMAD, inhibits the activation of SMAD 2/3 by the TGFBR. SMAD 7 is negatively regulated by microRNA-21 (miR-21). Active SMAD 2/3 binds the common SMAD (SMAD 4) and translocates to the nucleus to drive the transcription of genes encoding proteins that make up the extracellular matrix, proteins that are involved in epithelial to mesenchymal transition, and pro-fibrotic miRNA. The advanced glycation end products (AGEs) and angiotensin II (ANG 2) drive the activation of MAPKs that activate SMAD 2/3. Nitric oxide (NO) is involved in the inhibition of SMAD 2/3. Chrysin, rutin, and proanthocyanins inhibit the receptor for advanced glycation end products (RAGE), thus reducing the activation of MAPKs. In addition, propolis inhibits the activation of SMAD 2/3 by MAPKs. *Astragalus membranaceus* increases endothelial nitric oxide synthase (eNOS) and the level NO. A high level of NO inhibits the activation of SMAD 2/3. Rutin directly activates SMAD 7, leading to a reduction in activation of SMAD 2/3. *Carya cathayensis* inhibits miR-21, the negative modulator of SMAD7. *Phylanthus niruri*, propolis, bee venom, chrysin, ursolic acid, naringenin, genistein, rutin, betulinic acid reduce the level of TGFβ1, thus inhibiting the activation of TGFBR. Apigenin activates AMPK leading to a decrease in the level of TGFβ1.

The renoprotective effect of betulinic acid has been demonstrated in previous studies [252,253,254]. The antioxidant and anti-inflammatory properties of betulinic acid may be the leading cause of this effect. In particular, betulinic acid treatment has been shown to attenuate renal fibrosis in the CKD rat model by inhibiting levels of pro-fibrotic protein such as TGF-β, connective tissue growth factor (CTGF), fibronectin, collagen type I, and hydroxyproline, as well as enhancing renal structure and function (Figure 5) [255]. In addition, betulinic acid treatment suppressed diabetic renal fibrosis in STZ-induced diabetic rat kidneys and high glucose-treated glomerular mesangial cells by reducing the breakdown of IκBα and the activity of NF-κB and subsequently downregulation of fibronectin expression [256]. Betulinic acid therapy inhibited oxidative stress (MDA) and proinflammatory (IL-6, IL-1β, TNF-α) cytokines and improved cellular antioxidant enzymes (SOD and CAT) in STZ-induced DKD, the authors of this study related the protective role of betulinic acid to its effect on the pathway of AMPK/NF-κB/Nrf2 [257]. Similarly, but in another experimental model of membranous nephropathy, betulinic acid treatment enhanced the antioxidant status and relieved kidney inflammation through modulation in the expression of NF-κB, iNOS, TNF-α, Nrf2, HO-1, and NQO1 at both mRNA and protein levels [258]. Despite these exciting results, no clinical trial demonstrating the potential protective role of betulinic acid in the treatment of CKD patients has been conducted. Small bioactive compounds with anti-inflammatory and antioxidants properties against CKD are summarized in Table 2.

## 4. Conclusions

In this review, we have outlined the considerable benefits of many bioactive substances in the primary prevention of CKD, as well as in delaying the progression of CKD. These substances may also help manage and prevent two of the predominant common causes of CKD: diabetic nephropathy and renal fibrosis. Several biological pathways and mechanisms that contribute to the initiation and progression of CKD include the NF-κB pathway, mitochondrial activity, autophagy, and the TGF-β pathway. These substances may work by inhibiting oxidative stress, inflammation, and fibrosis, among other things, in the prevention and treatment of CKD. However, further research is needed to determine the particular mechanism through which these compounds exert their protective benefits. Although clinical trials have indicated that these natural antioxidants also can help manage and delay the progression of CKD, more prospective studies are needed, especially to determine the safety and side effects of these compounds. This paucity of clinical studies could be attributed to various causes, including a lack of finances, concerns about the compounds’ side effects and safety, and possibly a belief that pharmaceutical therapies are more vital.

## Figures and Tables

**Table 1:** Summary of clinical and experimental studies evaluating the protective effect and the possible mechanisms of different natural products against CKD. Decreased (↓) or increased (↑).

Natural Products	Type of Study	Therapeutic Effect	Major Findings	References
*Phylanthus niruri*	STZ-induced diabetic nephropathy (in vivo)	Antioxidant Anti-inflammatory Anti-apoptotic Anti-fibrotic	Kidney homogenate (TBARS ↓), (SOD, CAT and GPx ↑), (NF-κB-p65, Ikk-β, TNF-α, IL-1β and IL-6↓), (caspase-3, caspase-9, Bax ↓), (TGF-β1, VEGF, FGF-1 ↓),	[61]
*Carya Cathayensis*	- Mouse model of UUO (in vivo) - TGF-β1-treated mouse tubular epithelial cells (mTECs) (in vitro)	Anti-fibrotic	Collagens and α-SMA ↓ in the kidneys In vitro, fibrotic markers ↓ and miR-21 ↓ in TGF-β1-treated mouse tubular epithelial cells (mTECs). Smad7↑	[62]
*Flos A. manihot*	-Glomerulonephritis rabbit model - Diabetic nephropathy - Adriamycin-induced nephropathy - 417 patients with glomerular disease stages 1–2 CKD	Renoprotective agent Anti-apoptotic Antioxidant	Protein levels in urine ↓, apoptosis of podocytes↓, glomerulosclerosis ↓, and mesangial proliferation ↓ (kidneys)	[64,65,66,67,68,69]
*Astragalus membranaceus*	Human kidney proximal tubular epithelial cells (in vitro)	Anti-apoptotic and anti-inflammatory	H_2_O_2_ ↓, apoptosis↓, NF-κB ↓, TNF-α↓ (proximal epithelial cells)	[74]
	UUO rat kidney	Anti-fibrotic	Interstitial fibrosis ↓ eNOS ↑, ROS scavenging (kidney tissue)	[75]
	35 CKD patients (Stage 4 and 5, dose; 2.5 g/day)	Delayed kidney replacement	Maintain eGFR	[77]
	1804 CKD patients with diabetic nephropathy stage III–IV and case study (dose 30 g/day for 1 month)	Renal protective agent	Maintain serum BUN, SCr, CCr and urine protein↓, eGFR↑	[78,79]
	1323 CKD patients (all stages)	Renal protective agent	Blood hemoglobin and serum albumin ↑	[59]
*Astragalus mongholicus*	Diabetic nephropathy	Anti-inflammatory	Autophagy ↑, mTOR ↓, and PINK1/Parkin ↑	[80]
	UUO and puromycin aminonucleoside nephrosis rat model	Anti-fibrotic	mRNA TGF-β1 ↓, α-SMA ↓	[81,82,83]
*Vigna angularis*	STZ-induced diabetic nephropathy (in vivo)	Antioxidant Stimulate autophagy	Plasma GSH ↑, LC3B-II ↑, mRNA HO-1↓, p47phox ↓, plasma MDA↓, and mRNA MCP-1 ↓	[86,87]
Propolis	Aristolochic acids-induced nephropathy (in vivo)	Anti-fibrotic	Tubulointerstitial fibrosis ↓, TGF-β/Smad pathway ↓	[93]
Brazilian red propolis	5/6 nephroctomized rats	Antioxidant Anti-inflammatory	SCr ↓, proteinuria ↓ (serum and urine), infiltration of macrophages (kidney tissue) ↓	[98]
Indonesian propolis	UUO rat model	Antioxidant	Oxidative stress ↓, blood pressure ↓	[99]
Iranian propolis	STZ-induced diabetic nephropathy	Antioxidant	Serum MDA ↓, SOD↑, GPx ↑, improvement in histological architecture	[100]
Brazilian green propolis	148 CKD patients (type 2 diabetes) (Dose; 500 mg/day) Hemodialysis patients (250 mg/day, in capsules).	Renal protective agent Anti-inflammatory	Proteinuria ↓ (urine), inflammation ↓	[92,101]
Bee venom	UUO rat model	Anti-inflammatory Anti-fibrotic	mRNA TNF-α, IL-1β ↓, TGF-β1, FN, α-SMA ↓	[108]

STZ, streptoziticin; TBARS, thiobarbituric acid reactive substances; SOD, superoxide dismutase; CAT, catalase; GPx, glutathione peroxidase; NF-κB, nuclear factor kappa B; Ikk-β, IkappaB kinase; TNF-α, tumor necrosis factor alpha; IL-1β, interleukin -1 beta; IL-6, interleukin-6; Bax, BCL2-associated X protein; TGF-β1, transforming growth factor-beta1; VEGF, vascular endothelial growth factor; FGF-1, fibroblast growth factor; UUO, unilateral ureteral obstruction; α-SMA, smooth muscle alpha-actin; miR-21, microRNA 21; eNOS, endothelial nitric oxide synthase; H2O2, hydrogen peroxide; ROS, reactive oxygen species; eGFR, estimated glomerular filtration rate; BUN, blood urea nitrogen; Scr, serum creatinine; CCr, creatinine clearance; mTOR, mammalian target of rapamycin; PINK1/Parkin, PTEN-induced kinase 1/Parkin; GSH, glutathione; LC3B-II, light chain 3B-II; HO-1, heme oxygenase-1; p47phox, neutrophil cytosol factor 1; MDA, malondialdehyde; MCP-1, monocyte chemoattractant protein-1; Smad, small mothers against decapentaplegic; FN, fibronectin.

**Table 2:** Overview of studies investigating the protective effect and the possible mechanistic pathways of small bioactive compounds against CKD. Decreased (↓) or increased (↑).

Small Bioactive Compounds	Type of Study	Therapeutic Effect	Major Findings	References
Chrysin	Human mesangial cells (in vitro) Diabetic kidney disease (in vivo)	Anti-fibrotic	TGFβ1 and SMAD 2/3 ↓	[102]
	Adenine-induced CKD (in vivo)	Anti-inflammatory Antioxidant	Plasma TNF-α↓, SOD, CAT, GSH, TAC ↑ (renal homogenate)	[103]
Caffeic acid phenethyl ester (CAPE)	Lithium-induced renal toxicity (in vivo)	Antioxidant	Oxidative stress ↓, Antioxidant enzymes↑	[105]
Caffeic acid	STZ-induced diabetic nephropathy (in vivo)	Anti-inflammatory	IL-6, IL-1β, TNF-α, and MCP-1 ↓	[106]
Pinocembrin	STZ-induced diabetic nephropathy (in vivo)	Antioxidant	Oxidative stress and dyslipidemia↓	[107]
Berberine	- Cultured mouse podocytes (in vitro)	Anti-inflammatory	NF-κB ↓	[125]
	- STZ-induced diabetic nephropathy (in vivo)	Anti-inflammatory	TLR4, NF-κB ↓	[126]
	- Diabetic nephropathy (in vitro)	Prevent mitochondrial dysfunction	PGC-1α ↑, mitochondrial ROS ↓	[127]
	- 114 diabetic patients (type 2 diabetes) (0.4 g, 3 times a day)	Antioxidant	Urinary albumin/creatine ratio (UACR) ↓ and serum cystatin C (Cys C) ↓	[129]
Ursolic acid	- Adenine-induced kidney injury (in vivo)	Anti-fibrotic	TGF-β/Smad ↓, FN and collagen ↓	[140]
	- UUO mouse model (in vivo) and TGF-β1-treated HK-2 cells (in vitro)	Anti-fibrotic	Collagen 1, FN, α-SMA, snail1, slug, TGF-β1, and p-smad3 ↓	[141]
	- CKD nephroctomized mouse model (in vivo)	Anti-inflammatory	TGF-β, IL-6, and TNFα ↓	[142]
	Diabetic nephropathy (in vivo)	Anti-fibrotic Anti-inflammatory Antioxidant	ARAP1/AT1R ↓, renal inflammation, fibrosis, and oxidative stress↓	[143,144,145]
	Cultured murine podocytes (in vitro)	Activate autophagy	LC3II and Beclin1 ↑	[147]
Naringenin	- Obstructive nephropathy mice model and cell line (NRK52E)	Anti-fibrotic	Smad3 ↓, collagen I, α-SMA ↓ (renal tissue)	[164]
	- High cholesterol diet rat model	Antioxidant Anti-inflammatory	iNOS, TNF-α, IL-6, and NF-κB ↓ (renal tissue)	[167]
	- Diabetic rat model	Antioxidant Anti-fibrotic Anti-inflammatory	MDA ↓, (SOD, CAT, GSH ↑), TGF-β1 and IL-1 ↓ (renal tissue)	[168]
	Diabetic mice model and NRK-52E cells	Renoprotective	PPARs-CYP4A-20-HETE pathway ↑	[169]
Apigenin	Human endothelial cells (in vitro)	Antioxidant	Oxidative stress ↓, p38/MAPK pathway ↓	[184]
	Diabetic rat model	Activation of autophagy Anti-inflammatory	Mitochondrial dysfunction ↓, oxidative stress ↓, Sirt3 ↓, CD38↓ Nrf2/HO-1 ↑, NF-κB ↓	[187,188]
Genistein	Diabetic rat model and rat mesangial cells exposed to high glucose	Anti-fibrotic	TGF-β1, p-Smad3, collagen IV ↓ (renal tissue)	[203,204]
	Cultured murine podocytes exposed to high glucose	Activation of autophagy	Autophagy ↑, mTOR signaling pathway ↓	[206]
	Diabetic mouse model	Antioxidant Anti-inflammatory	Cox-2, MCP-1, TNF-α and NF-κB ↓, Nrf2, GPx, SOD and HO-1↑ (renal tissue)	[207]
	Isolated mononuclear cells from hemodialysis patients	Anti-inflammatory	TNF-α ↓ (mononuclear cells)	[208]
Rutin	Adenine-induced CKD rat animal model	Anti-fibrotic Antioxidant	Tubulointerstitial fibrosis ↓, HO-1↓, PLA-2 ↓ (renal tissue)	[217]
	5/6 nephrectomy and UUO rat models	Anti-fibrotic	Renal fibrosis ↓, TGFβ1-Smad signaling pathway↓	[218,219]
	Endothelial cells of glomeruli exposed to hyperglycemia	Antioxidant	Nrf2 ↑, RhoA/ROCK pathway ↓	[221]
	Alloxan-induced diabetic nephropathy in rats	Anti-fibrotic Antioxidant	TGF- β1↓, podocin ↑, GRP78 and CHOP ↓, ketoacidosis and fibrosis ↓. (renal tissue)	[222,223]
	STZ-induced diabetic nephropathy in rats	Anti-fibrotic	Collagen IV, laminin, TGF-β1, p-Smad 2/3, (CTGF) ↓ (renal tissue)	[224]
Proanthocyanidin	Mice subjected to ischemia/reperfusion (I/R)	Anti-fibrotic Anti-inflammatory	TGF-β, IL-6 and TNFα ↓, HMGB1/TLR4/p65/TGF-β1 signaling pathway ↓ (renal tissue)	[232]
	UUO mice and primary renal tubular epithelial cells (PTEC), normal rat kidney fibroblast (NRK-49F)	Anti-fibrotic	C3/HMGB1//TGF-β1 ↓ (renal tissue)	[233]
	STZ-induced diabetic nephropathy in rats	Anti-inflammatory	AGEs/RAGE axis ↓ (renal tissue)	[234]
	STZ-induced diabetic nephropathy in rats	Antioxidant Anti-inflammatory Anti-apoptotic	MDA ↓, IL-6 ↓, GSH ↑, SOD, Nrf2 ↑, GRP78), p-ERK, and caspase 12 ↓ (renal tissue)	[235,236,237,238]
Betulinic acid	Adenine-induced CKD rat animal model	Anti-fibrotic	TGF-β, (CTGF), FN, collagen type I, and hydroxyproline ↓ (renal tissue)	[255]
	STZ-induced diabetic nephropathy in rats and glomerular mesangial cells treated with high glucose level	Anti-inflammatory Anti-fibrotic	IκBα, NF-κB pathway ↓, FN expression ↓	[256]
	STZ-induced diabetic nephropathy in rats	Anti-inflammatory Antioxidant	IL-6, IL-1β, TNF-α ↓ (serum and kidney tissue) AMPK ↑/NF-κB↓/Nrf2 ↑	[257]
	Membranous nephropathy rat model	Anti-inflammatory Antioxidant	NF-κB ↓, iNOS ↓, TNF-α ↓, Nrf2 ↑, HO-1↑, and NQO1 ↑ (renal tissue)	[258]

TGFβ1 and SMAD 2/3, transforming growth factor-beta1 and small mothers against decapentaplegic 2/3; TNF-α, tumor necrosis factor alpha; SOD, superoxide dismutase; CAT, catalase; GSH, glutathione; TAC, total antioxidant capacity; TNF-α, tumor necrosis factor alpha; IL-1β,interleukin -1 beta; IL-6, interleukin-6; MCP-1, monocyte chemoattractant protein-1; TLR4, Toll-like receptor 4; NF-κB, nuclear factor kappa B; PGC-1α, peroxisome proliferator-activated receptor-gamma coactivator -1α; FN, fibronectin; α-SMA, smooth muscle alpha-actin; snail1, zinc finger protein SNAI1; slug, zinc finger protein SNAI2; p-smad3, phosphorylated small mothers against decapentaplegic 3; ARAP1/AT1R; angiotensin II type 1 receptor-associated protein/angiotensin II type 1 receptor; LC3II, light chain 3-II; Beclin1, BECN1; α-SMA, smooth muscle alpha-actin; iNOS, inducible nitric oxide synthase; MDA, malondialdehyde; PPARs, peroxisome proliferator-activated receptors; 20-HETE, 20-hydroxyeicosatetraenoic acid; CYP4A, cytochrome P450 4a; MAPK, mitogen-activated protein kinases; Sirt3, sirtuin-3; CD38, cluster of differentiation 38; Nrf2, nuclear factor erythroid 2-related factor 2; HO-1, heme oxygenase-1; mTOR, mammalian target of rapamycin; MCP-1,monocyte chemoattractant protein-1; GPx, glutathione peroxidase; Cox-2; cyclooxygenase-2; PLA-2, phospholipases A2; RhoA, ras homolog gene family, member A; ROCK, Rho-associated protein kinase; GRP78, glucose-regulated protein78; CHOP, C/-EBP homologous protein; CTGF, connective tissue growth factor; HMGB1, high-mobility group box 1; p65, transcription factor p65; C3, complement 3; AGEs, advanced glycation end products; RAGE, receptor for advanced glycation end products; p-ERK, phosphorylated extracellular signal-regulated kinase; IκBα; nuclear factor of kappa light polypeptide gene enhancer in B-cells inhibitor, alpha; AMPK, AMP-activated protein kinase; NQO1, NAD(P)H: quinone oxidoreductase 1.
